# Drawing-Induced Crimp Formation and Wettability of Four-Lobed Side-by-Side PBT/PET Bicomponent Fibers

**DOI:** 10.3390/polym17182529

**Published:** 2025-09-18

**Authors:** Xinkang Xu, Pei Feng, Zexu Hu, Jiazheng Wang, Qianchun Xu, Chongchang Yang

**Affiliations:** 1 College of Mechanical Engineering, Donghua University, Shanghai 201620, China; 1212014@mail.dhu.edu.cn (X.X.); huzexu@dhu.edu.cn (Z.H.); 2231056@mail.dhu.edu.cn (J.W.); 2231200@mail.dhu.edu.cn (Q.X.); ycc@dhu.edu.cn (C.Y.); 2 Engineering Research Center of Advanced Textile Machinery, Donghua University, Shanghai 201620, China; 3 State Key Laboratory of Fiber Material Modification, Donghua University, Shanghai 201620, China

**Keywords:** PBT/PET side-by-side fibers, draw-induced crimp, profiled cross-section, four-element viscoelastic model, wettability

## Abstract

PBT/PET side-by-side bicomponent fibers form helical crimp structures under thermal or mechanical stress, though the mechanism behind mechanically induced crimping remains unclear. In this study, four-lobed cross-sectional PBT/PET side-by-side bicomponent fibers were produced and subjected to drawing from 1.6 to 4.0 times at 80 °C to induce crimping. Increasing draw ratios significantly enhanced fiber tenacity (from 0.64 to 3.91 cN/dtex) and reduced crimp radius (from 2.05 mm to 0.64 mm). A predictive crimp curvature model integrating Denton’s crimp theory and a four-element viscoelastic model was established, with corrected results achieving an R^2^ of 0.9951. Additionally, four-lobed fibers showed better wettability, with a static contact angle 3.56° lower than that of circular fibers. This work provides theoretical guidance for high-performance self-crimping fiber design.

## 1. Introduction

Self-crimping fibers possess superior elasticity, structural bulk, dye affinity, and dimensional stability. These properties make them highly attractive for a wide range of textile applications, including apparel, home furnishings, and performance-oriented sports fabrics [1]. Among them, side-by-side bicomponent elastic fibers—such as PBT/PET—exploit the distinct physical and thermal properties of two polymers to generate internal stress imbalances under thermal or mechanical stimuli, leading to the spontaneous formation of spiral crimp structures [2,3]. These crimped morphologies not only enhance fiber bulkiness and resilience but also significantly affect the dynamic mechanical behavior [4], tactile comfort, and overall functional performance of fabrics [5,6]. Therefore, elucidating the crimp formation mechanisms and developing controllable strategies has become a key focus in the field of functional fiber design [7].

Theoretical studies on crimp formation trace back to Timoshenko’s classical thermal bending model for bimetallic strips, which demonstrated that mismatched thermal expansion coefficients cause structural curvature—laying the groundwork for multi-component fiber crimp modeling [8]. Building upon this, Brand and Backer introduced a helical spring model to simulate the three-dimensional crimp behavior of bicomponent fibers [9]. Subsequently, Denton developed a generalized curvature model applicable to arbitrary cross-sectional geometries and component distributions, significantly enhancing the predictive capability of self-crimping fiber systems [10]. More recently, Khadse et al. reported temperature-responsive PBT bicomponent fibers, in which thermally triggered differential shrinkage enabled adaptive crimping behavior, though the role of viscoelastic effects was not considered [11]. Further, Zhang et al. combined numerical simulation with experimental validation to investigate helix curvature in bicomponent helical fibers, providing quantitative insights into the effects of strain mismatch, modulus ratio, and processing conditions [12]. However, these studies primarily focused on thermally induced crimping, whereas mechanically induced (e.g., draw-triggered) crimp formation remains relatively underexplored—particularly from a theoretical and quantitative perspective [13].

In recent years, increasing attention has been paid to the effects of melt-spinning parameters on the crimp characteristics of side-by-side bicomponent fibers. For example, filaments composed of high- and low-intrinsic-viscosity PET exhibit optimal self-crimping behavior at a 50:50 component ratio, primarily due to the combined effects of differential shrinkage and molecular orientation during thermal treatment [14,15,16,17]. Although several reviews have summarized the classification, formation mechanisms, and structural optimization strategies of bicomponent fiber systems [18,19,20], most existing research remains empirical, lacking in-depth theoretical modeling of draw-induced crimp formation.

In addition to mechanical performance, the functional behavior of elastic fibers in wettability has also become a research focus, especially in the context of performance textiles. Meanwhile, growing interest has emerged in the moisture management performance of profiled (non-circular) fibers. It has been shown that fiber cross-sectional geometry and fineness play a crucial role in capillary action, water vapor transport, and drying efficiency [21]. Multi-lobed and cross-shaped fibers demonstrate superior moisture-wicking capabilities compared to conventional round fibers, making them especially suitable for functional and sports textiles [22]. SUN et al. reported the development of moisture-wicking and fast-drying knitted fabrics using PTT/PET bicomponent filaments with grooved, shaped cross-sections. The fabrics exhibited excellent sweat absorption and evaporation capabilities, achieved purely through structural design without chemical finishing. These findings underscore the synergistic benefits of bicomponent fiber architecture and fabric morphology in enhancing moisture management [23]. However, that study was limited to bicomponent fibers composed of two round filaments and did not explore more complex geometries, such as multi-lobed cross-sections. Comparable mechanisms have also been highlighted in microfluidic studies, where serpentine channel geometries were shown to promote droplet breakup and enhance liquid spreading [24,25]. Such insights provide a useful analogy for fabric-scale moisture transport, reinforcing the importance of curvature and flow path geometry in governing wettability.

In response to these gaps, this study investigates four-lobed PBT/PET side-by-side bicomponent fibers fabricated via melt spinning. By applying drawing at 80 °C under various draw ratios, helical crimp structures are induced and systematically characterized. A novel analytical model, combining Denton’s crimp theory and a four-element viscoelastic framework, is developed to quantitatively interpret the draw-induced crimping mechanism from a non-thermal perspective, with predictions validated against experimental measurements. The central research question is therefore whether draw-induced viscoelastic shrinkage mismatch can quantitatively explain the crimp formation during drawing in profiled bicomponent fibers and how the wettability differs between four-lobed and circular fibers as a result of cross-sectional geometry. Addressing this question provides both theoretical and experimental insights for the structural design of high-performance, self-crimping bicomponent fibers with enhanced functional attributes.

## 2. Materials and Methods

### 2.1. Materials and Equipment

Experimental materials were polyethylene terephthalate (PET) chips with an intrinsic viscosity of 0.68 ± 0.010 dL/g (provided by Hengli Petrochemical Co., Ltd. Suzhou, China) and polybutylene terephthalate (PBT) chips with an intrinsic viscosity of 1.20 ± 0.015 dL/g (provided by Wuxi Xingsheng New Materials Technology Co., Ltd. Wuxi, China). The corresponding melt flow rates were 9.6 g/10 min (260 °C/2.16 kg) for PET and 16.5 g/10 min (250 °C/2.16 kg) for PBT. Before use, both PET and PBT chips were dried in a vacuum oven for 10 h to ensure adequate moisture removal. Key fiber preparation equipment included a multi-component melt-spinning machine (custom-designed, Jiangxi Donghua Machinery Co., Ltd. Fuzhou, China) with independently controlled spin-pack temperatures, a four-lobed spinneret (custom-designed, Donghua University, Shanghai, China) for forming the fiber cross-section, and a heated multi-roll draw frame (custom-designed, Donghua University, Shanghai, China).

### 2.2. Fiber Preparation

#### 2.2.1. Melt Spinning

**Figure 1:**
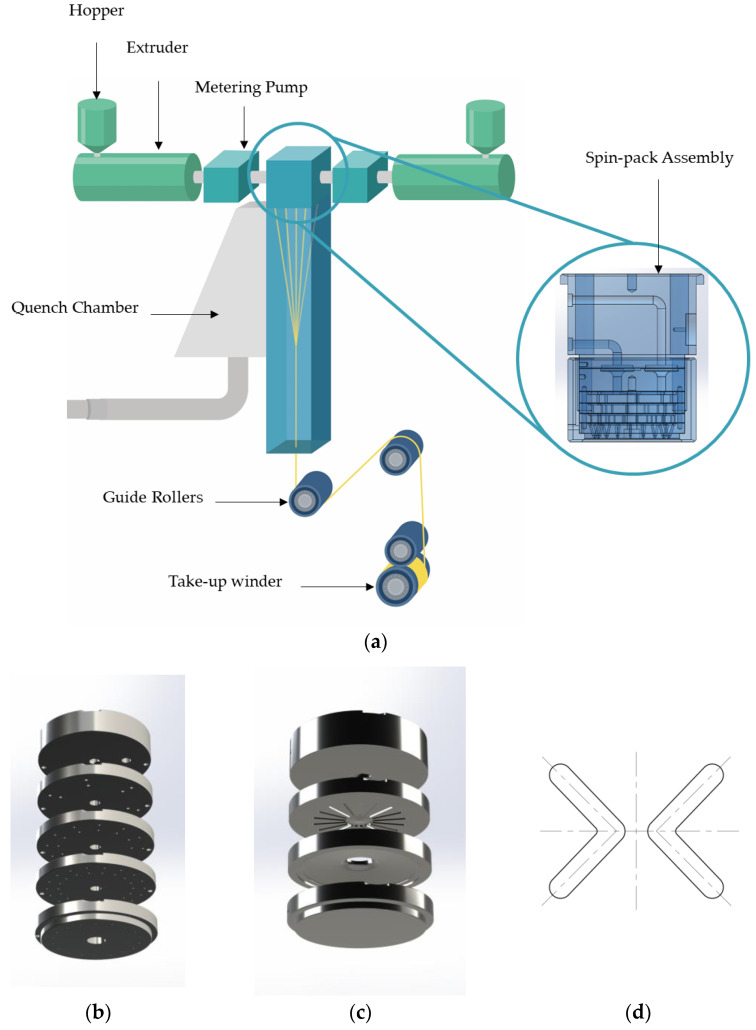
Melt-spinning line and spin-pack assemblies. (**a**) Process flow of the side-by-side bicomponent spinning; (**b**) side-by-side spin-pack assembly; (**c**) single-component spin-pack assembly; (**d**) four-lobed spinneret microhole.

Dried PET and PBT chips were separately added into two extrusion systems of a melt-spinning machine. The polymers were melted by heating zones 1–4 of the screw extruder and extruded at a constant speed by a gear pump through a specialized four-lobed spinneret to form initial fibers. The experimental setup for preparing four-lobed PBT/PET side-by-side bicomponent fibers is shown in Figure 1a, and the spin-pack assembly is shown in Figure 1b.

The four-lobed spin-pack assembly contained 18 pairs of symmetric side-by-side orifices, as shown in Figure 1d. In each pair, one orifice extruded PET and the other extruded PBT, resulting in 18 bicomponent filaments in total. Each lobe was designed with a width of 0.10 mm and a length of 0.45 mm. Since both PBT and PET are polyester-based polymers, they exhibit partial compatibility. Immediately after extrusion, the polymer streams undergo die swell due to viscoelastic relaxation, causing the filaments to expand and come into closer contact at the interface. As the two polymer streams travel along the spinning path in a high-temperature viscous or semi-molten state, interfacial molecular chains can diffuse and entangle, leading to physical adhesion between the PET and PBT components.

The melt experienced shear stress in the spinneret microholes, exhibited die swell upon exiting the capillaries, and quickly cooled. Under take-up, the filaments were attenuated, ultimately collected by a take-up winder. Spinning zone temperatures and key processing parameters are listed in Table 1. Notably, due to significant differences in thermomechanical properties of PBT and PET, fibers composed of these materials easily developed non-uniform shrinkage and internal stress distributions during subsequent drawing and thermal treatments, laying the foundation for crimp formation.

In addition, the single-component PET and PBT filament yarns (220 dtex/36f) used for the creep experiments were produced in the laboratory using the same melt-spinning platform and polymer batches as those employed for the bicomponent fibers, and the single-component spin-pack assembly is shown in Figure 1c. The processing route followed that of the bicomponent fibers, and no post-drawing was applied.

#### 2.2.2. Drawing and Heat Setting

**Figure 2:**
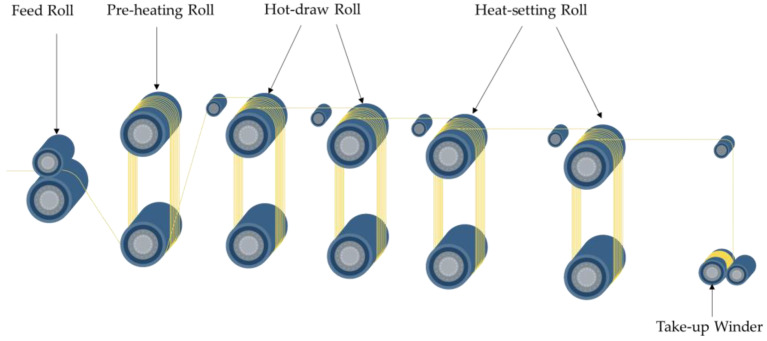
Schematic of the heated multi-roll draw frame showing feed (Roll 1), preheat (Roll 2), hot-draw (Roll 3, 4), heat-set (Roll 5, 6), and take-up; draw ratio set by the velocity ratio of Roll 4 to Roll 3.

Initial fibers obtained from spinning underwent drawing to induce crimp formation. The process diagram of the drawing machine is shown in Figure 2. On a heated multi-roll draw frame, the wound fibers were unwound and sequentially fed through one feed roll and five pairs of draw rolls. Fibers were preheated and predrawn between Rolls 2–3. The overall draw ratio (DR) was imposed by the linear-speed ratio of Roll 4 to Roll 3 (DR = V_4_/V_3_). Heat setting was performed at fixed length between Rolls 5–6; Rolls 1–5 were maintained at 80 °C, while Roll 6 was set at 25 °C to enable cooling. Upon exiting the heated zone the filaments were air-cooled to stabilize the crimp. Finally, yarns were taken up on the winder. The tested draw ratios were 1.6, 2.2, 2.8, 3.4, and 4.0, and the process parameters are listed in Table 2.

### 2.3. Fiber Testing Methods

Fiber Fineness Measurement. Fiber linear density was determined by the skein method (100 m hank) using a skein-length tester (YG086, Nantong Sansi Electromechanical Technology Co., Ltd., Nantong, China) and a high-precision balance (ME104E, Mettler Toledo, Switzerland; readability ≤ 0.1 mg). Ten hanks were prepared per condition (n = 10), weighed, and the mean ± SD was used to calculate fineness.

Fiber Cross-Sectional Morphology (SEM). Cross-sections were prepared by embedding in epoxy, trimming, and polishing, then sputter-coating with 5 nm Pt/Pd. Images were acquired on a GeminiSEM 300 (Carl Zeiss AG, Oberkochen, Germany) at 3 kV and a working distance of 7.4 mm.

Mechanical Property Tests. Single-filament tenacity, elongation at break, and initial modulus were measured using a YG029 single-fiber tester (Laizhou Electronic Instrument Co., Ltd., Laizhou, China) under standard laboratory conditions (20 ± 2 °C, 65 ± 4% RH). Unless otherwise specified, gauge length = 250 mm, crosshead speed = 250 mm/min, and a pretension of 0.05 cN/dtex were applied; n = 10 filaments per draw ratio, and results are reported as mean ± SD.

Fiber Tension Measurement. In-line draw tension was recorded with a SCHMIDT ETPX-200 tension meter (Hans Schmidt & Co. GmbH, Waldkraiburg, Germany; resolution 1 cN) mounted between Roll 3 and Roll 4. Data were sampled after steady state; for each draw ratio, n = 10 independent trials were performed and averaged (mean ± SD).

Creep Behavior Tests: Constant-load creep of single-component fibers (pure PET and pure PBT) was measured on a CTM2050 electronic universal testing machine (Xie Qiang Instrument Manufacturing Co., Ltd. Shanghai, China) at 20 ± 2 °C and 65 ± 4% RH. Each specimen was preloaded with a tension of 0.05 cN/dtex prior to testing. A constant tensile load of 50 cN was applied; gauge length = 250 mm; strain was recorded at 10 Hz for 300 s to characterize viscoelastic behavior. Test specimens were laboratory-prepared filament yarns (220 dtex, 36 filaments) spun on the same platform as the bicomponent fibers and without post-drawing; n = 3 per material.

Crimp Radius Measurement. Helical crimp images were acquired using a VHX-X1 digital microscope (Keyence Corporation, Osaka, Japan). For each draw condition, n = 10 filaments were randomly selected and imaged at calibrated magnification with a built-in scale bar. For each filament, three consecutive turns were analyzed. The inscribed circle of each helical turn was extracted and fitted by least-squares circle fitting to obtain the crimp radius *R*, which was then averaged. Results are reported as mean ± SD.

Static contact angles were measured on single fibers using a JY-PHb contact-angle analyzer (Chende Yote Co., Ltd., Chengde, China) by the sessile-drop method (5 μL ultrapure water). Droplet–fiber interaction was recorded by CCD, and the equilibrium angle was obtained by the tangent method from left/right profiles. Test conditions were 20 ± 2 °C and 65 ± 4% RH; n = 8 fibers per group.

## 3. Results and Discussion

### 3.1. Fiber Fineness and Cross-Sectional Morphology

The measured fiber fineness before and after drawing is presented in Table 3.

**Figure 3:**
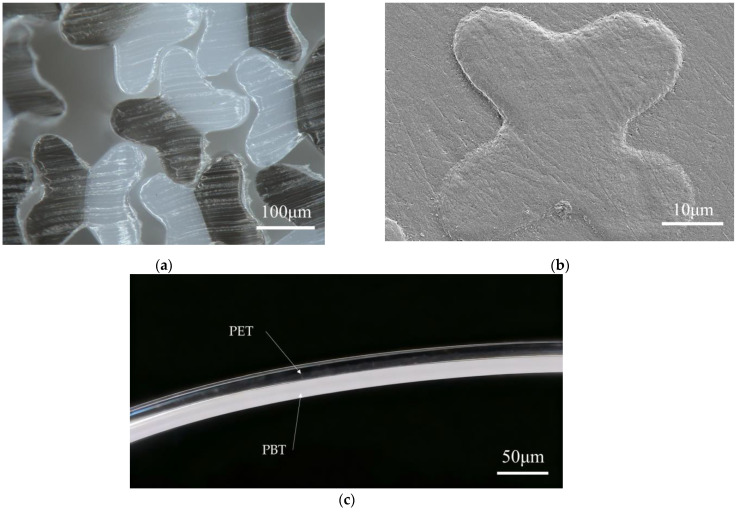
Cross-sectional and longitudinal morphology of four-lobed PBT/PET filaments: (**a**) free-fall, unwound filaments collected directly below the spinneret (PET light, PBT dark; optical micrograph, 100×); (**b**) wound at 1000 m/min (SEM, 2000×); (**c**) longitudinal view of fibers at DR = 1.6 (optical micrograph, 200×).

SEM and optical microscopy were used to examine the cross-sectional morphology of the fibers (Figure 3). The side-by-side bicomponent filaments exhibit a symmetric four-lobed profile with clearly separated lobes; in Figure 3a, the lighter domains correspond to PET and the darker ones to PBT. This brightness difference arises because PET was prepared from bright-grade chips with high transparency, making its cross-section appear lighter under optical microscopy. The interlobal angles between adjacent PET–PBT lobes are slightly smaller than those between opposing lobes belonging to the same component, reflecting differences in interfacial and surface tensions. Compared with the free-fall (unwound) filaments in Figure 3a, the filaments in Figure 3b display a markedly smaller apparent cross-section, attributable to in-line extensional draw-down during winding at a take-up speed of 1000 m/min. Moreover, longitudinal observation of fibers drawn at DR = 1.6 (Figure 3c) confirmed that the PET component occupies the outer side, while the PBT component lies on the inner side.

### 3.2. Tensile Mechanical Properties

**Figure 4:**
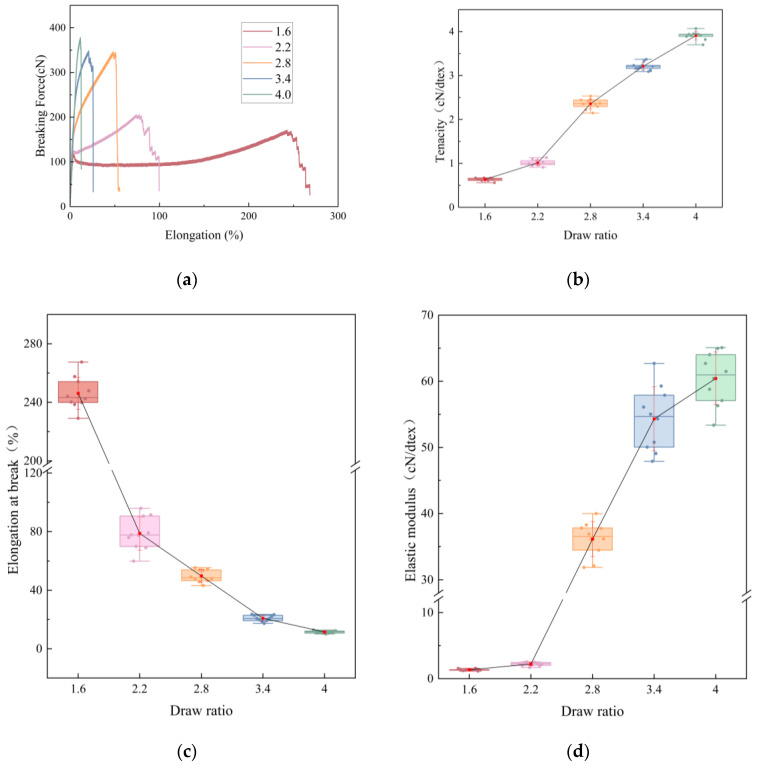
Tensile properties vs. draw ratio at 20 ± 2 °C/65 ± 4% RH: (**a**) Representative force–elongation curve; (**b**) Variation of tenacity with draw ratio; (**c**) Variation of elongation at break with draw ratio; (**d**) Variation of initial modulus with draw ratio.

Mechanical properties evolve systematically with draw ratio. Tenacity rises from 0.64 ± 0.03 cN/dtex at DR = 1.6 to 1.01 ± 0.07, 2.36 ± 0.11, 3.21 ± 0.09, and 3.91 ± 0.10 cN/dtex at DR = 2.2, 2.8, 3.4, and 4.0, respectively, while elongation at break drops from 246.15 ± 11.05% to 77.78 ± 9.58%, 49.78 ± 4.21%, 20.83 ± 2.07%, and 11.44 ± 0.86%. The initial modulus correspondingly increases from 1.33 ± 0.13 to 2.22 ± 0.27, 36.14 ± 2.63, 54.31 ± 4.84, and 60.42 ± 3.98 cN/dtex. Representative force–elongation curves are provided in Figure 4a, while the monotonic shifts—higher strength/stiffness with reduced ductility—are visualized with box-and-whisker plots overlaid with mean ± SD in red, where boxes denote Q1–Q3, the center line is the median, whiskers span the minimum–maximum range, and dots are individual values, as shown in Figure 4b–d. These results indicate that drawing substantially enhances the strength and stiffness of the fibers but at the cost of reduced ductility. This variation in mechanical properties is attributed to the increased molecular chain orientation and crystallinity during the drawing process, which leads to a more orderly and compact fiber structure, thus improving load-bearing capacity (strength and stiffness) while decreasing plastic deformability. The accumulation of internal stress differences between the two components due to drawing is expected to serve as a key driving force for the formation of crimped structures.

Normality tests (Shapiro–Wilk) confirmed that all mechanical property data sets followed a normal distribution (*p* > 0.05). Levene’s test showed that the assumption of homogeneity of variance held for tenacity (*p* = 0.227) but was violated for elongation at break (*p* = 0.011) and initial modulus (*p* < 0.001). Accordingly, one-way ANOVA was used for tenacity, while Welch’s ANOVA was adopted for elongation at break and modulus. The results demonstrated that draw ratio had a highly significant effect on all three properties: tenacity (F = 2738.9, *p* < 0.001, η^2^ = 0.996), elongation at break (Welch’s F (4, 19.5) = 1276.1, *p* < 0.001, η^2^ = 0.993), and initial modulus (Welch’s F (4, 18.9) = 1175.4, *p* < 0.001, η^2^ = 0.987). The very large effect sizes indicate that almost all of the variance in mechanical performance can be attributed to the applied draw ratio.

### 3.3. Crimp Radius and Crimping Mechanism

**Figure 5:**
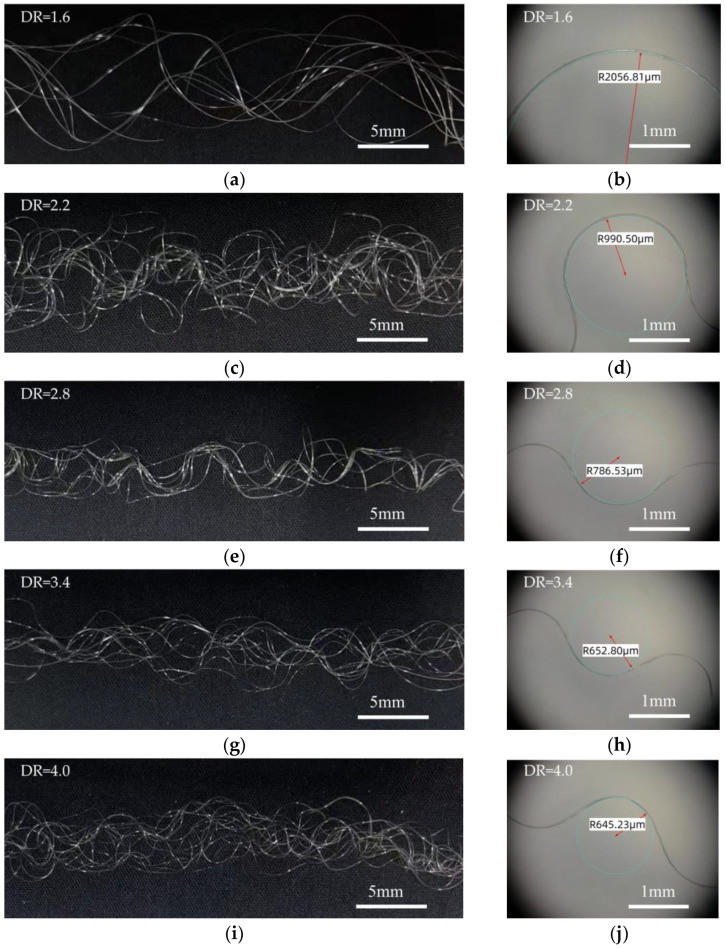
Crimp morphology and radius of curvature at different draw ratios (PET on the outer side, PBT on the inner side): (**a**,**b**) Draw ratio of 1.6; (**c**,**d**) Draw ratio of 2.2; (**e**,**f**) Draw ratio of 2.8; (**g**,**h**) Draw ratio of 3.4; (**i**,**j**) Draw ratio of 4.0.

The crimp morphology of fibers under different draw ratios is shown in Figure 5. During drawing and subsequent cooling, the two components undergo differential shrinkage, resulting in the spontaneous formation of crimp structures upon stress release. In the curved fiber structure, PET is positioned on the outer side, while PBT occupies the inner side due to its higher shrinkage. As the draw ratio increases, the crimp frequency increases (crimp pitch decreases) and the curvature becomes more pronounced. This indicates that higher draw ratios introduce greater internal stress differences, leading to tighter and more pronounced crimp structures. Further quantitative analysis shows that, with the increase in draw ratio, the fiber crimp radius gradually decreases and tends to stabilize.

**Figure 6:**
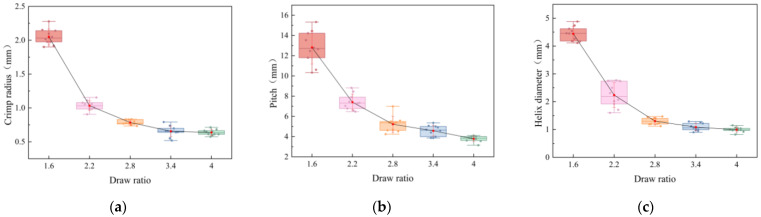
Variation of helix geometry with draw ratio: (**a**) Crimp radius; (**b**) Helix pitch; (**c**) Helix diameter.

To further quantify the geometric characteristics of the helical structures, the helix pitch, helix diameter, and crimp radius were extracted from microscope images and analyzed across draw ratios. As shown in Figure 6, all three parameters exhibited monotonic reductions with increasing draw ratio. Specifically, the pitch decreased from 12.71 mm at DR = 1.6 to 3.78 mm at DR = 4.0, the helix diameter shrank from 4.44 mm to 1.00 mm, and the crimp radius declined from 2.05 mm to 0.64 mm. These results indicate that higher draw ratios intensify the internal stress mismatch between PET and PBT components, producing tighter and more compact helical structures.

The crimp geometry parameters, including crimp radius, pitch, and helix diameter, were all normally distributed (Shapiro–Wilk, *p* > 0.05). However, Levene’s test indicated heterogeneity of variances across draw ratios (*p* < 0.05). Therefore, Welch’s ANOVA was applied for these variables. The results revealed highly significant differences among draw ratios (*p* < 0.001), with very large effect sizes (η^2^ > 0.92). This quantitative analysis complements the crimp radius results and provides a comprehensive description of draw-induced helix geometry.

In addition to the intrinsic thermal shrinkage difference between the two polymers, the higher intrinsic viscosity of PBT (1.20 dL/g vs. 0.68 dL/g for PET) also contributes to its shrinkage behavior. A higher intrinsic viscosity implies longer chain length and greater entanglement density, which hinder molecular relaxation during spinning and drawing. Consequently, the PBT phase retains more residual orientation and internal stress. Upon unloading or subsequent heating, these stresses are released as additional contraction, thereby amplifying the effective shrinkage difference relative to PET.

#### 3.3.1. Theoretical Basis of Crimp Formation

**Figure 7:**
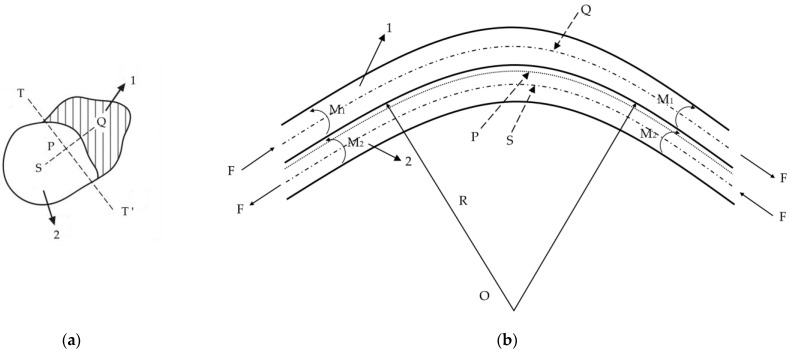
Geometric definitions for Denton’s crimp curvature model: (**a**) cross-section; (**b**) longitudinal view [10].

The spontaneous crimping of side-by-side bicomponent fibers requires two essential conditions: good interfacial compatibility between the two components and a significant difference in their intrinsic free shrinkage behavior [26]. For a side-by-side bicomponent fiber of arbitrary cross-sectional shape, Denton’s model integrates cross-sectional geometric parameters and material properties to compute the resulting crimp curvature. Let the centroid of the entire fiber cross-section be point P and the centroids of components 1 and 2 be points Q and S, respectively. The perpendicular bisector of line QS passes through point P and is defined as the TT’ axis, as shown in Figure 7. The cross-sectional areas of the two components are *A*_1_ and *A*_2_, and the distances from their respective centroids to point P are $u_{1}$ and $u_{2}$. The second moments of area of the cross-sections, taken about axes through Q and S and parallel to TT’, are *I*_1_ and *I*_2_, respectively. The instantaneous elastic moduli of components 1 and 2 are denoted by *E*_e_ and *E*_b_.

Due to differences in free shrinkage capabilities between the two components, the high-shrinkage component (assumed as component 2) exerts a compressive force *F* on the low-shrinkage component 1. At the same time, component 1 exerts an equal and opposite tensile force on component 2. Together, these forces form a force couple, which induces bending stress within the fiber and results in internal bending moments *M*_1_ and *M*_2_ for each component. Based on moment equilibrium, an equation can be established equating the moment of this internal force couple with the fiber’s resistance to bending. Solving this balance yields the fiber’s crimp curvature radius R. As shown in Equation (1), Denton’s model expresses the crimp curvature as a function of material and geometric parameters.

(1) $$\rho =\frac{1}{R}=\frac{A_{2}A_{0}u_{2}\Delta }{A_{0}I_{0}+\left(m-1\right)\left[A_{2}I_{2P}-\frac{1}{m}A_{1}I_{1P}-\frac{m-1}{m}A_{2}^{2}u_{2}^{2}\right]}$$

In the formula, $A_{0}=A_{1}+A_{2}$, $I_{1P}=I_{1}+A_{1}u_{1}^{2}$, $I_{2P}=I_{2}+A_{2}u_{2}^{2}$, $I_{0}=I_{1P}+I_{2P}$, $m=E_{b}/E_{e}$, Δ is the free shrinkage difference.

The crimp curvature of the fiber depends on several key parameters: the difference in free shrinkage ratio between the two components, the ratio of their elastic moduli, their cross-sectional areas, and their second moments of area. The free shrinkage difference refers to the strain difference exhibited by the two components in the absence of external constraint, and it serves as one of the primary driving forces for inducing the bending deformation that leads to crimp formation.

#### 3.3.2. Draw-Induced Shrinkage Difference

**Figure 8:**
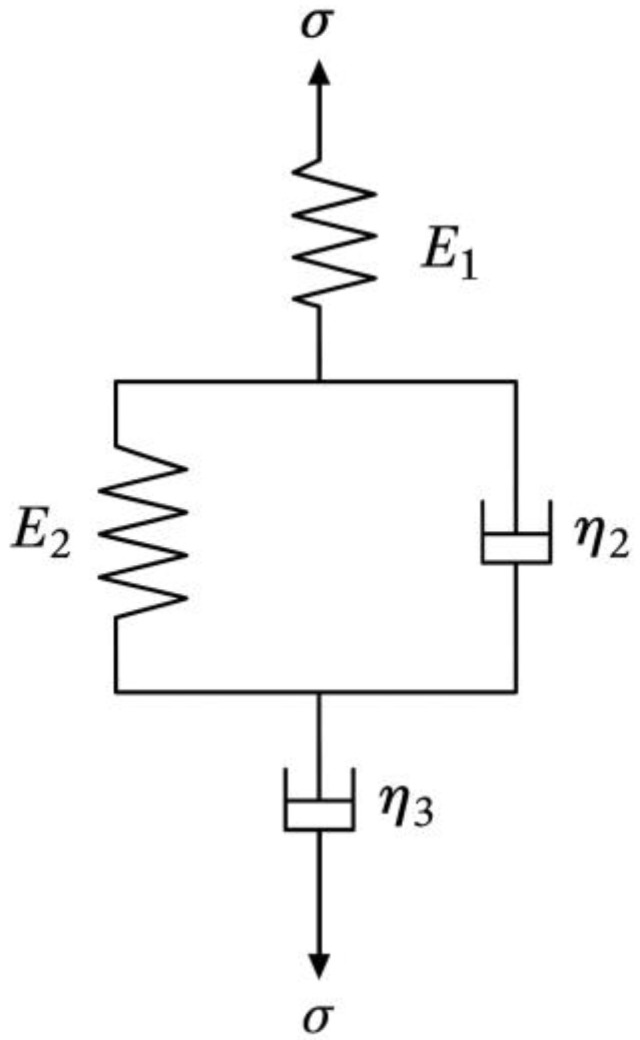
Four-element linear viscoelastic model used to fit PET/PBT creep behavior [27].

Fibers composed of long-chain polymer aggregates exhibit time-dependent deformation characteristics due to complex internal rearrangements during tensile loading. In addition to the drawing of the main molecular chains, there is gradual rupture of secondary bonds, progressive chain alignment, and reorganization of the internal fiber structure. As a result, PET and PBT fibers exhibit both elastic solid and viscous fluid behavior—known as viscoelasticity. To simulate this viscoelastic behavior, classical linear viscoelastic models such as the Maxwell model, Kelvin model, three-element model, and four-element model are commonly used [27]. In this study, the four-element model was selected to fit the creep behavior of PBT and PET fibers, as shown in Figure 8, as it captures the three critical stages during fiber drawing: instantaneous elastic deformation, delayed elastic deformation, and irreversible viscous flow [28].

The four-element model consists of the following components: a standalone Hookean spring (with modulus *E*_1_) to represent instantaneous elastic strain; a Kelvin unit (composed of a spring with modulus *E*_2_ in parallel with a dashpot of viscosity $\eta_{2}$) to simulate delayed elastic strain; and a pure viscous dashpot (viscosity $\eta_{3}$) connected in series with the combined parallel unit to model unrecoverable viscous (plastic) deformation.

When a constant tensile stress $\sigma_{c}$ is applied, the total creep strain $\varepsilon \left(t\right)$ of the four-element model can be expressed as the sum of strains from each component: the instantaneous elastic strain $\varepsilon_{1}$, the time-dependent delayed elastic strain $\varepsilon_{2}$, and the accumulated viscous flow strain $\varepsilon_{3}$ resulting from continuous external loading, as shown in Equation (2).

(2) $$\varepsilon \left(t\right)=\frac{\sigma_{c}}{E_{1}}+\frac{\sigma_{c}}{E_{2}}\left(1-e^{-t/\tau_{k}}\right)+\frac{\sigma_{c}}{\eta_{3}}t$$

The retardation time constant, $\tau_{k}=\eta_{2}/E_{2}$, characterizes the material’s viscoelastic behavior. Each of the three strain components has distinct physical significance: $\varepsilon_{1}$ represents the instantaneous elastic strain that fully recovers immediately upon unloading and is time-independent; $\varepsilon_{2}$ denotes the time-dependent delayed elastic strain that gradually recovers after unloading—$\tau_{k}$ being the time required to reach ($1-e^{-1}$) of its final value; and $\varepsilon_{3}$ corresponds to the continuously growing viscous flow strain, which remains as permanent residual deformation after unloading.

**Figure 9:**
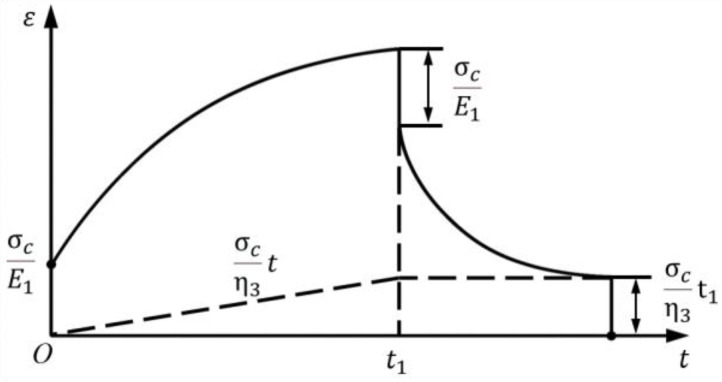
Predicted creep and recovery response of the four-element model under constant load and unloading at *t*_1_ [27].

As shown in Figure 9, as time progresses, $\varepsilon_{1}$ remains constant, $\varepsilon_{2}$ approaches saturation, and $\varepsilon_{3}$ continues to increase. When unloading occurs at time *t* = *t*_1_, $\varepsilon_{1}$ instantly recovers, $\varepsilon_{2}$ decays gradually to zero, while $\varepsilon_{3}$ remains unchanged, as shown in Figure 9. If no postdrawing heat setting is applied, the delayed elastic strain $\varepsilon_{2}$ can fully recover over time, leaving only the irreversible viscous strain $\varepsilon_{3}$ as the permanent deformation. However, if the fiber undergoes heat setting at a fixed length after drawing, the delayed strain $\varepsilon_{2}$ is gradually converted into plastic deformation during the heating process. For simplicity, it is assumed that all delayed strain is converted into permanent deformation during heat setting, meaning that only $\varepsilon_{1}$ recovers upon unloading.

Furthermore, assuming perfect bonding between the two components during drawing (i.e., no interfacial slip and equal strain between PBT and PET), the total tensile force *F* acting on the fiber is shared between the PET component (*F*_1_) and the PBT component (*F*_2_). At the unloading instant *t* = *t*_1_, the stress ratio between the two components is determined by their respective elastic moduli: $\sigma_{c1}/\sigma_{c2}=E_{e}\left(t_{1}\right)/E_{b}\left(t_{1}\right)$. The respective instantaneous elastic recovery strains of the PET and PBT components are denoted as $\varepsilon_{h1}\left(t_{1}\right)$ and $\varepsilon_{h2}\left(t_{1}\right)$. Accordingly, the free shrinkage difference between the two components induced by unloading after drawing is defined as: $\Delta \varepsilon =\varepsilon_{h2}\left(t_{1}\right)-\varepsilon_{h1}\left(t_{1}\right)$. This Δε, caused by differential elastic recovery after unloading, serves as one of the key driving forces for the formation of crimped fiber structures.

#### 3.3.3. Thermally Induced Shrinkage Difference

The thermal shrinkage behavior of fibers is a crucial factor in the design of traditional self-crimping fibers. When exposed to dry or humid heat, the originally oriented polymer chains undergo relaxation and reorganization, resulting in irreversible shrinkage deformation [29]. For PBT/PET side-by-side fibers, the two components exhibit different degrees of free shrinkage under heating. The difference in thermal shrinkage between the two components is defined as Δ*S* = *S*_2_ − *S*_1_.

Typically, the value of Δ*S* can be obtained through fiber thermal shrinkage experiments, which primarily consider the influence of temperature. Unlike draw-induced crimping, which forms immediately upon unloading, thermally induced crimping is a gradual process, usually stabilizing about 5 min after the onset of heating [30]. Moreover, the elastic modulus of fiber materials decreases with increasing temperature during thermal shrinkage, complicating the accurate prediction of crimp radius. To simplify the model, it is assumed that the ratio of elastic moduli between the two components remains constant until shrinkage is complete. Under this assumption, the contributions of draw-induced and thermally induced crimping to the overall crimp curvature can be additively calculated. Based on this model, the expression relating fiber crimp curvature to draw ratio, temperature, modulus ratio, and cross-sectional parameters is given by Equation (3).

(3) $$\rho =\frac{1}{R}=\frac{A_{2}A_{0}u_{2}(\Delta \varepsilon +\Delta S)}{A_{0}I_{0}+\left(h\left(t_{1}\right)-1\right)\left(A_{2}I_{2P}-\frac{1}{h\left(t_{1}\right)}A_{1}I_{1P}-\frac{h\left(t_{1}\right)-1}{h\left(t_{1}\right)}A_{2}^{2}u_{2}^{2}\right)}$$

In the formula, $h\left(t_{1}\right)=E_{b}\left(t_{1}\right)/E_{e}\left(t_{1}\right)$, elastic modulus ratio at time *t*_1_.

This equation enables the analysis of the variation in fiber crimp radius under different drawing and thermal treatment conditions.

From the perspective of the theoretical framework in Equation (3), this viscosity effect is reflected through the material-dependent parameters rather than the geometric ones: (i) the modulus ratio $h\left(t_{1}\right)$, which is influenced by viscosity-dependent relaxation times; (ii) the draw-induced free shrinkage difference Δε, since the viscoelastic constants (*E*_1_, *E*_2_, $\eta_{2}$, $\eta_{3}$) scale with intrinsic viscosity; and (iii) the thermally induced shrinkage Δ*S*, whose effective value under short hot-roll heating depends on chain mobility and stress-retention capacity. Together, these factors explain why the PBT side consistently occupies the inner position of the crimped structure and why higher PBT viscosity enhances the crimping tendency.

#### 3.3.4. Calculation of Shrinkage Difference

**Figure 10:**
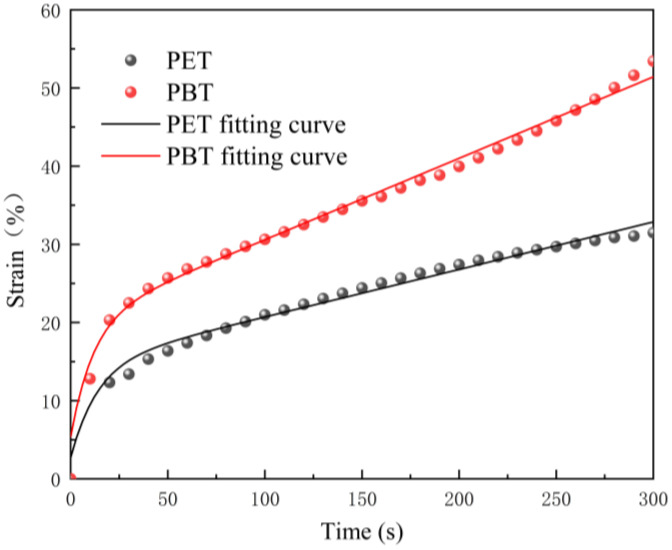
Scatter plot and fitted curves of constant-load creep data for laboratory-spun PET and PBT yarns.

To quantitatively calculate the free shrinkage difference between the two components under different drawing conditions, constant-load creep experiments were conducted on pure PET and pure PBT filament yarns to characterize their viscoelastic behavior. Laboratory-spun PET and PBT filament yarns (220 dtex/36f) were used as test specimens, as shown in Figure 10.

It should be noted that both the single-component and bicomponent fibers were prepared under the same spinning conditions and collected at a take-up speed of 1000 m/min. The single-component PET and PBT fibers used for creep experiments and the bicomponent fibers prior to postdrawing were therefore in the same as-spun state. Consequently, using the creep behavior of single-component fibers to parameterize the viscoelastic model provides a reasonable basis for simulating the deformation state of the bicomponent fibers during subsequent drawing.

The non-linear least-squares method in MATLAB (R2020a) was used to fit the creep equations of the four-element viscoelastic model for both fiber types, extracting the model parameters *E*_1_, *E*_2_, $\eta_{2}$, and $\eta_{3}$. The goodness of fit (*R*^2^) for both materials exceeded 0.9788, indicating that the four-element model effectively describes the creep behavior of PET and PBT fibers. The fitted viscoelastic parameters are listed in Table 4.

By substituting the experimentally obtained fiber cross-sectional geometric parameters, the average draw tensions under different draw ratios, and the viscoelastic parameters of the single-component fibers derived from Table 4 into Denton’s crimp model, the theoretical curvature of draw-induced crimping can be calculated and compared with the experimentally measured crimp radii.

**Figure 11:**
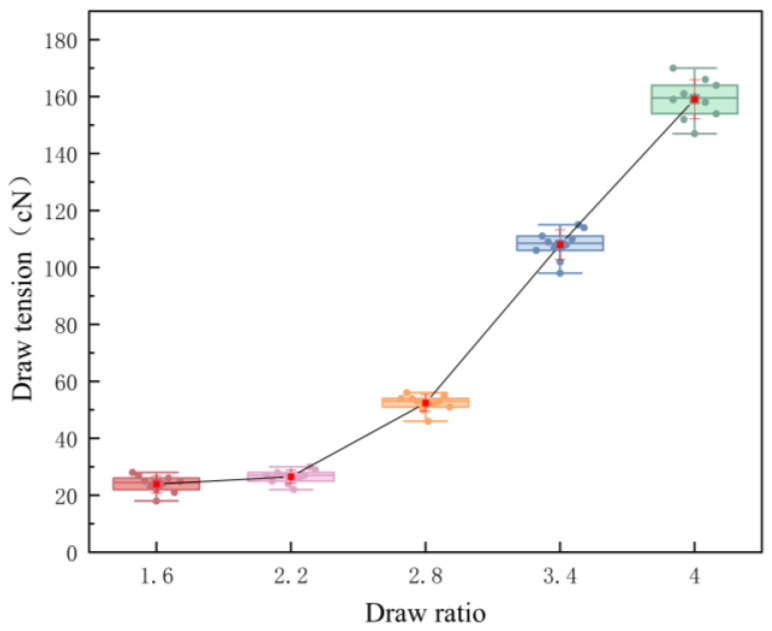
In-line draw tension at different draw ratios, measured between Roll 3 and Roll 4.

**Figure 12:**
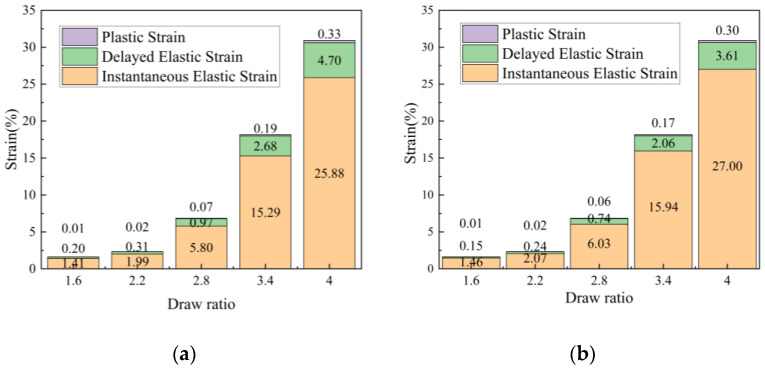
Decomposed tensile strain components across draw ratios from the viscoelastic fit: (**a**) PET; (**b**) PBT (instantaneous elastic, delayed elastic, and plastic).

Based on the previously established model and assuming that the delayed elastic deformation during the drawing process is fully converted into plastic deformation during heat setting, the draw tensions from Figure 11 and the viscoelastic parameters from Table 4 were used to calculate the strain decomposition (instantaneous elastic strain, delayed elastic strain, and plastic strain) and the free shrinkage difference Δ*ε* for the two components under different draw ratios. The decomposed strain components for PET and PBT are illustrated in Figure 12. The tensile shrinkage differences of the resulting fibers are listed in Table 5.

**Figure 13:**
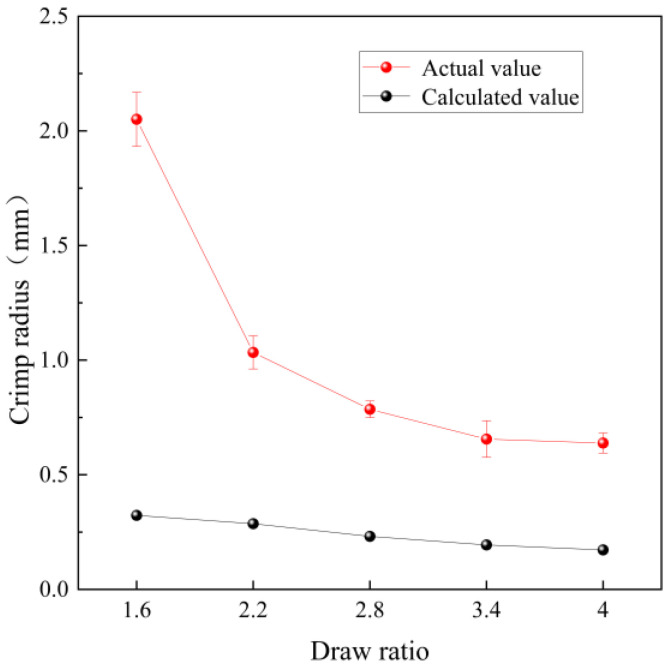
Crimp radius: measured values vs. model predictions using literature boiling water shrinkage.

According to literature data, the boiling water shrinkage of PET fiber is approximately 4.9%, while that of PBT fiber is about 13% [30], resulting in a shrinkage difference of 8.1%. Based on the curvature equation and the tensile shrinkage differences from Table 5, the crimp radii at different draw ratios were calculated and compared with the experimental values, as shown in Figure 13. The calculation results show that, as the draw ratio increases, the free shrinkage difference between PET and PBT components increases significantly. When the model-predicted crimp curvature is converted to crimp radius and compared with the measured values, the trends are generally consistent. However, the initial predicted values from the model tend to be lower than the experimental measurements.

The discrepancy arises primarily because the shrinkage difference parameters used in the model are derived from boiling water shrinkage values reported in the literature, while the actual thermal conditions during the drawing process (such as heating by 80 °C hot rolls) differ from the boiling water environment. This leads to deviations in actual shrinkage behavior from the idealized assumptions. To address this, we introduce an empirical correction factor *C* into the original model to reconcile discrepancies between theory and experiment, as given in Equation (4):

(4) $$\rho =\frac{1}{R}=\frac{A_{2}A_{0}u_{2}(\Delta \varepsilon +C\Delta S)}{A_{0}I_{0}+\left(h\left(t_{1}\right)-1\right)\left(A_{2}I_{2P}-\frac{1}{h\left(t_{1}\right)}A_{1}I_{1P}-\frac{h\left(t_{1}\right)-1}{h\left(t_{1}\right)}A_{2}^{2}u_{2}^{2}\right)}$$

A least-squares fit of the experimental data in MATLAB (R2020a) produced the optimal expression for the correction factor *C*, given in Equation (5): with *x* representing the draw ratio.

(5) $$C=-0.0954x^{2}+0.5364x-0.4609$$

The correction parameter *C* introduced in Equation (5) does not merely serve as a mathematical fitting term; rather, it reflects the discrepancy between the idealized shrinkage data (obtained from literature-reported boiling water shrinkage values) and the actual thermal–mechanical history experienced during the drawing process. Specifically, the 80 °C hot-roll treatment involves a much shorter heating duration and different heat-transfer conditions compared to boiling water, leading to variations in chain relaxation, orientation recovery, and shrinkage behavior. Therefore, *C* can be regarded as an empirical factor that incorporates the combined effects of (i) the deviation in shrinkage kinetics under different heating environments, (ii) the influence of the four-lobed cross-section on internal stress redistribution, and (iii) the coupling between stretch-induced and thermally induced shrinkage during drawing and heat setting. Although introduced phenomenologically, *C* provides a practical means to reconcile theoretical predictions with experimental observations, and its magnitude may offer insights into the extent of structural relaxation and stress mismatch under specific processing conditions.

**Figure 14:**
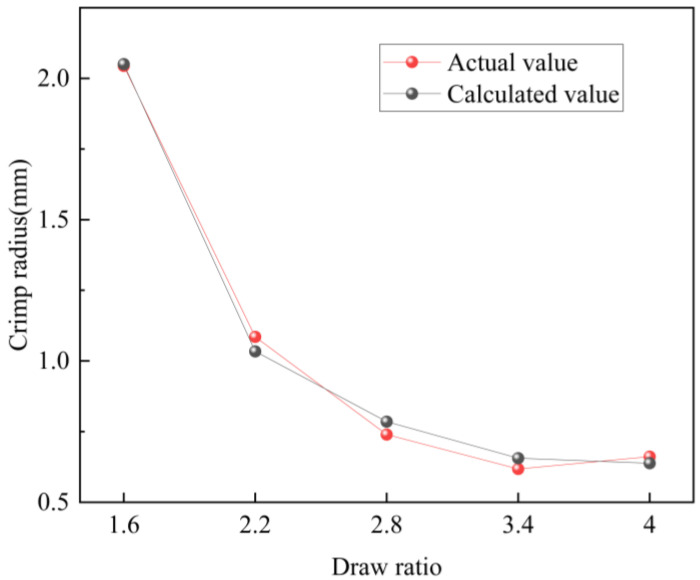
Crimp radius: measured values vs. predictions corrected by empirical factor *C(x)*.

After introducing this parameter, the agreement between the model predictions and experimental measurements improved significantly, as shown in Figure 14. The coefficient of determination *R*^2^ increased to 0.9951, indicating excellent model performance. Despite this correction, the model still exhibits several limitations in practical application:

Deviation in parameter values: The model relies on literature-reported boiling water shrinkage data (approximately 4.9% for PET and 13% for PBT). However, the actual thermal history during the drawing process (such as brief heating on an 80 °C hot roll) differs from boiling water conditions, potentially resulting in discrepancies between the assumed and actual shrinkage behavior, thus introducing error. Boiling water shrinkage was adopted here because it is a commonly measured parameter, enabling practical estimation of crimp curvature without additional tests.

Coupling between drawing and thermal shrinkage: In practice, draw-induced and thermally induced crimping are not entirely independent. Partial thermal shrinkage may occur during drawing, and subsequent heat setting and ambient temperature changes also affect internal stress relaxation. Since the model introduces only a single correction parameter, it cannot fully represent this complex coupling. Therefore, the final crimp morphology results from the combined effects of tensile stress release and thermal shrinkage, which may be additive or counteractive. Future work should focus on constructing more sophisticated coupled models and validating them with further experiments.

### 3.4. Wettability Performance

**Figure 15:**
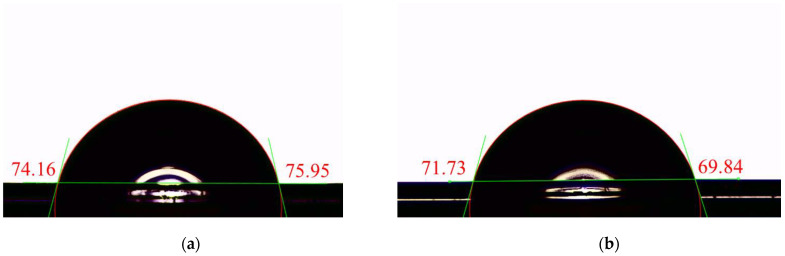
Static contact angle on single fibers: (**a**) Circular fiber; (**b**) Four-lobed fiber.

The wettability performance of fibers with different cross-sections was characterized using a single-fiber contact angle test, as shown in Figure 15. The results tabulated in Table 6 showed that the average static contact angle of fibers with a four-lobed cross-section was 71.28°, lower than that of circular cross-section fibers, which was 74.84°—a reduction of approximately 3.56°.

The static contact angle measurements for circular and four-lobed fibers were normally distributed (Shapiro–Wilk, *p* > 0.05), and Levene’s test confirmed homogeneity of variances (*p* = 0.545). A Student’s *t*-test revealed that four-lobed fibers exhibited a significantly lower contact angle (71.3° ± 2.0°) than circular fibers (74.8° ± 2.2°, *p* = 0.024, Cohen’s d = 1.26). This large effect size indicates that the profiled cross-section substantially improves single-fiber wettability, attributable to the increased specific surface area and enhanced local curvature. While these single-fiber results provide a reliable indicator of surface wettability, they cannot fully capture fabric-level moisture management (e.g., wicking and drying), which requires further textile-scale investigation. Nevertheless, the present findings confirm the effectiveness of cross-sectional profile design in enhancing fiber-scale wettability, and future work will extend this analysis to fabrics while considering potential artifacts such as fiber rotation.

## 4. Conclusions

This study integrates theoretical modeling and experiments to investigate drawing-induced crimp formation in four-lobed PBT/PET side-by-side bicomponent fibers and to evaluate single-fiber wettability (static contact angle). A predictive model was developed by integrating Denton’s crimp theory with a four-element viscoelastic framework, enabling quantitative analysis of non-thermal crimp formation mechanisms.

Experimental results showed that increasing the draw ratio from 1.6 to 4.0 significantly improved fiber strength (0.64 to 3.91 cN/dtex) and modulus (1.33 to 60.42 cN/dtex), while reducing the crimp radius from 2.05 to 0.64 mm. These changes stem from enhanced molecular orientation and growing internal stress differences between components. The model predictions closely matched experimental data (*R*^2^ = 0.9951) after applying a correction factor. Notably, the reduction in crimp radius with draw ratio is primarily driven by the increasing mismatch in instantaneous elastic strain between PET and PBT during drawing and thermal stabilization.

Static contact-angle measurements showed that four-lobed fibers had a lower contact angle (71.28° vs. 74.84°), reflecting better single-fiber wettability due to their profiled four-lobed cross-section and the resulting increase in specific surface area and local curvature. We note that these results are limited to the single-fiber scale; comprehensive evaluation of moisture management and fast-drying behavior requires future fabric-level studies.

Overall, this work provides theoretical and practical guidance for designing self-crimping fibers and tuning single-fiber wettability via structural engineering and controlled processing. Fabric-level moisture management should be assessed with textile-scale tests in future work. In addition, the present study has several limitations. The shrinkage difference (Δ*S*) was taken from literature data rather than measured under the actual processing conditions, and an empirical correction factor was introduced to reconcile model predictions with experiments. Furthermore, the coupling between draw-induced and thermal shrinkage was only phenomenologically represented. Future work will address these issues by directly measuring Δ*S* under hot-roll conditions, conducting DMA tests over a temperature range to refine viscoelastic parameters, extending the wettability evaluation to fabric-scale experiments, and performing a more detailed sensitivity analysis to propagate parameter uncertainties into the predicted crimp radius. These efforts will provide a more comprehensive validation and broaden the applicability of the proposed model.

## Figures and Tables

**Table 1:** Melt-spinning parameters for side-by-side PBT/PET with a four-lobed spinneret.

Item		PET Component Parameters	PBT Component Parameters
Extruder barrel temperatures	Zone 1 (°C)	270	250
	Zone 2 (°C)	290	270
	Zone 3 (°C)	293	278
	Zone 4 (°C)	293	285
Melt temperature upstream of the gear pump (°C)		293	285
Melt temperature downstream of the gear pump (°C)		293	285
Spinneret block temperature (°C)		290	290
Ambient temperature (°C)		25	25
Polymer throughput rate (g·min^−1^·hole^−1^)		1.09	1.05
Take-up speed (m/min)		1000	1000

**Table 2:** Drawing and heat-setting parameters for side-by-side PBT/PET fibers.

Draw Ratio	Roll 1 Speed (m/min)	Roll 2 Speed (m/min)	Roll 3 Speed (m/min)	Roll 4 Speed (m/min)	Roll 5 Speed (m/min)	Roll 6 Speed (m/min)	Take-Up Speed (m/min)
1.6	10	10	10	16	16	16	16
2.2	10	10	10	22	22	22	22
2.8	10	10	10	28	28	28	28
3.4	10	10	10	34	34	34	34
4.0	10	10	10	40	40	40	40

**Table 3:** Measured fiber fineness before and after drawing, average of 10 tests.

Initial Fineness (dtex)	Draw Ratio	Fineness (dtex)
394.10	1.6	266.83
	2.2	212.22
	2.8	142.88
	3.4	110.33
	4	95.94

**Table 4:** Fitted four-element viscoelastic parameters and goodness of fit for PET and PBT.

Fiber Type	*E*_1_ (cN/dtex)	95% CI	*E*_2_ (cN/dtex)	95% CI	$\eta_{2}$ (cN·s/dtex)	95% CI	$\eta_{3}$ (cN·s/dtex)	95% CI	*R* ^2^
PET	8.3868	[6.43, 10.35]	1.9066	[1.76, 2.05]	26.0542	[21.75, 30.36]	373.8221	[364.26, 383.38]	0.9788
PBT	4.2353	[3.40, 5.07]	1.5352	[1.49, 1.58]	17.7848	[16.66, 18.91]	218.1426	[215.59, 220.69]	0.9892

**Table 5:** Draw-induced free shrinkage difference between PBT and PET vs. draw ratio.

Draw Ratio	Δ*ε* (%)
1.6	0.048
2.2	0.075
2.8	0.233
3.4	0.641
4	1.119

**Table 6:** Static contact angle statistics (mean ± SD) and 95% confidence intervals for circular and four-lobed fibers.

Fiber Cross-Section	Number of Measurements	Mean Contact Angle (°)	Standard Deviation	95% Confidence Interval for the Mean (°)	
				Lower Limit	Upper Limit
Circle	8	74.84	2.23	72.98	76.70
Four-lobed	8	71.28	1.96	69.64	72.92

## Data Availability

The original contributions presented in the study are included in the article, further inquiries can be directed to the corresponding author.

## References

[1] Hufenus R., Yan Y., Dauner M., Kikutani T. (2020). Melt-spun fibers for textile applications. Materials.

[2] Wan A., Xu T., Yao C., Gao L. (2024). Effect of heat treatment on the mechanical properties of PBT/PET yarn. J. Ind. Text..

[3] Yu J., Li X., Ji H., Zhang Y., Chen K. (2021). Evaluation of the crimp formability of side-by-side PLA/PTT bicomponent fibers. Text. Res. J..

[4] Wang F., Gu F., Xu B. (2013). Elastic strain of PTT/PET self-crimping fibers. J. Eng. Fibers Fabr..

[5] Souissi M., Khiari R., Zaag M., Meksi N., Dhaouadi H. (2021). Effect of the morphology of polyester filaments on their physical properties and dyeing performances. Polym. Bull..

[6] Wan A., Xu Z., Yao C., Ma P. (2025). Preparation and properties of heat–moisture adaptive elastic seamless sports underwear. Text. Res. J..

[7] Gernhardt M., Peng L., Burgard M., Jiang S., Förster B., Schmalz H., Agarwal S. (2018). Tailoring the morphology of responsive bioinspired bicomponent fibers. Macromol. Mater. Eng..

[8] Timoshenko S.S. (1925). Analysis of bi-metal thermostats. J. Opt. Soc. Am..

[9] Brand R.H., Backer S. (1961). Mechanical principles of natural crimp of fiber. Text. Res. J..

[10] Denton M.J. (1982). The crimp curvature of bicomponent fibres. J. Text. Inst..

[11] Khadse N., Ruckdashel R., Macajoux S., Sun H., Park J.H. (2022). Temperature-responsive PBT bicomponent fibers for dynamic thermal insulation. Polymers.

[12] Zhang X., Xu Y., Li H., Li Y., Zhang Y., Zhao T., Zeng Y. (2023). Investigation on the Helix Curvature of Bicomponent Helical Fibers: Numerical Simulation and Experimental Validation. Phys. Fluids.

[13] Luo J. (2010). Self-Crimping Structure and Properties of PTT/PET Side-by-Side Bicomponent Fibers. Master’s Thesis.

[14] Xiang G., Hua H., Gao Q., Guo J., Zhang X., Wang X. (2022). Fabrication and properties of self-crimp side-by-side bicomponent filaments composed of polyethylene terephthalates with different intrinsic viscosity. Fibres Text. East. Eur..

[15] Abbasi M., Kotek R. (2019). Effects of drawing process on crimp formation-ability of side-by-side bicomponent filament yarns produced from recycled, fiber-grade and bottle-grade PET. J. Text. Inst..

[16] Gao F., Sun Y., Xiao S., Chen W., Lu W. (2022). Microstructure and properties of polyester composite fibers with different drafting ratios. J. Text. Res..

[17] Rwei S.P., Lin Y.T., Su Y.Y. (2005). Study of self-crimp polyester fibers. Polym. Eng. Sci..

[18] Zhu S., Meng X., Yan X., Chen S. (2021). Evidence for bicomponent fibers: A review. e-Polymers.

[19] Naeimirad M., Zadhoush A., Kotek R., Esmaeely Neisiany R., Nouri Khorasani S., Ramakrishna S. (2018). Recent advances in core/shell bicomponent fibers and nanofibers: A review. J. Appl. Polym. Sci..

[20] Lamberger Z., Zainuddin S., Scheibel T., Lang G. (2023). Polymeric janus fibers. ChemPlusChem.

[21] Das B., Das A., Fangueiro R., de Araujo M. (2008). Effect of fibre diameter and cross-sectional shape on moisture transmission through fabrics. Fibers Polym..

[22] Xu B., Xu B., Shi Z., Lu C., Hu Z., Cheng Y., Zhu M., Jiang L., Liu H. (2024). Continuous homogeneous thin liquid film on a single cross-shaped profiled fiber with high off-circularity: Toward quick-drying fabrics. Adv. Mater..

[23] Sun S., Peng M., Liu J., Liu Y., Zhou W., Dai H., Tan L., Yu J., Li G. (2025). A novel moisture-wicking and fast-drying functional bicomponent fabric. Fibers Polym..

[24] Der O., Bertola V. (2020). An experimental investigation of oil–water flow in a serpentine channel. Int. J. Multiph. Flow.

[25] Wu Z., Sundén B. (2019). Liquid–liquid two-phase flow patterns in ultra-shallow straight and serpentine microchannels. Heat Mass Transf..

[26] Liu Y.L., Yuan R.C., Yu J.Y., Liu L., Li X. (2025). Preparation and properties of PA6/PBT side-by-side bicomponent elastic fibers. J. Donghua Univ. (Nat. Sci.).

[27] Yu W.D., Chu Y.C. (2002). Textile Physics.

[28] Möginger B. (1993). The determination of a general time creep compliance relation of linear viscoelastic materials under constant load and its extension to nonlinear viscoelastic behavior for the Burger model. Rheol. Acta.

[29] Wang N., Sun R.J., Lai K. (2003). Study on the effect of heat treatment on thermal shrinkage of polyester filament yarn. J. Donghua Univ. (Nat. Sci.).

[30] Zhang Y. (2006). Study on Component Compatibility in PBT/PET Blends. Master’s Thesis.

